# Unresectable metastatic colorectal cancer in fit patients – a practical algorithm of treatment sequencing from the Brazilian Group of Gastrointestinal Tumours (GTG)

**DOI:** 10.3332/ecancer.2023.1544

**Published:** 2023-05-02

**Authors:** Renata D’Alpino Peixoto, Anelisa K Coutinho, Gabriel Prolla, Rui F Weschenfelder, Rachel Riechelmann

**Affiliations:** 1 Centro Paulista de Oncologia (Oncoclínicas), São Paulo 04538-132, Brazil; 2 Clínica AMO (DASA), Salvador 41950-640, Brazil; 3 Oncoclínicas, Porto Alegre 90570-020, Brazil; 4 Hospital Moinhos de Vento, Porto Alegre 90035-000, Brazil; 5 AC Camargo Cancer Center, São Paulo 01509-001, Brazil

**Keywords:** algorithm, metastatic colorectal cancer, colon cancer treatment

## Abstract

Recent advances in biomarker-driven therapies have changed the landscape of unresectable metastatic colorectal cancer (mCRC) and brought not only access issues but also difficulties for the treating physician (especially generalist oncologists) in choosing the most suitable treatment for each individual patient. This manuscript proposes an algorithm developed by The Brazilian Group of Gastrointestinal Tumours with the aim of bringing easy-to-follow steps in the management of unresectable mCRC. The algorithm is based on evidence for fit patients to facilitate therapeutic decisions in the clinical practice and assumes that there are no access and resource limitations.

According to GLOBOCAN 2020, approximately 10 million cancer deaths occur worldwide per year and colorectal cancer (CRC) accounts for 9.4% of them, representing the second leading cause of cancer mortality [1]. Thereby, metastatic CRC (mCRC) poses an immense public health challenge and deserves further attention. Recent advances in biomarker-driven therapies changed the landscape of unresectable mCRC and brought not only access issues but also difficulties for the treating physician (especially generalist oncologists) in choosing the most suitable treatment for each individual patient [2].

Unfortunately, most guidelines are not straightforward when it comes to deciding the treatment sequencing of mCRC in algorithms. Most of them focus mainly on the first-line settings while others have yet not incorporated recent advances [3–5]. The Brazilian Group of Gastrointestinal Tumours (GTG) recognises the difficulty of bringing easy-to-follow steps in the management of unresectable mCRC and proposes an ideal algorithm based on evidence for fit patients to facilitate therapeutic decisions in the clinical practice, although limitations in depicting all possible clinical scenarios need to be recognised, such as maintenance and locoregional therapies (Figure 1). Our algorithm was built on the assumption that there are no access and resource limitations. Therefore, it should not be considered a regulatory guideline. We recognise that in most parts of the world many of the options recommended are not available. In this case, one should move to the next step in this algorithm. In addition, whenever possible, patients should be encouraged to participate in clinical trials.

Approximately 3%–5% of mCRC have microsatellite instability (MSI-high)/deficient mismatch repair and are currently best treated with first-line immunotherapy. The highest level of evidence we have so far is with pembrolizumab in monotherapy [6]. However, the combination of ipilimumab and nivolumab is also promising and approved in many countries [7]. After progression to first-line immunotherapy in MSI-H, we prefer to offer a second-line doublet (fluoropyrimidine with irinotecan or oxaliplatin) with bevacizumab if raf murine sarcoma viral oncogene homolog B1 (BRAF) wild-type, especially if rat sarcoma (RAS) mutated or right-sided tumours. Recent data suggest certain resistance to anti-EGFR agents in MSI-H tumours [8]. However, it is also possible to offer a doublet with anti-EGFR in the second-line setting if RAS wild-type and left-sided since data of resistance to anti-EGFR in MSI-H tumours is still scarce. Since MSI-H mCRC may coexist with BRAF V600E mutations, a BRAF inhibitor combined with cetuximab may be offered as second-line regimen when this is the case [9]. Further lines of therapy for MSI-H tumours follow the same rules as for microsatellite stable (MSS) mCRC. When the patient progresses on second-line doublet therapy with bevacizumab and maintains good performance status, a further decision is based on molecular profiling. After that, trifluridine-tipiracil (TFD/TPI) with bevacizumab (preferable) or regorafenib are options [10, 11].

For patients with MSS tumours harbouring a RAS mutation, our recommended first-line option is a doublet (FOLFOX, CAPOX or FOLFIRI) with bevacizumab, although eventually a triplet (FOLFOXIRI) with bevacizumab may be used in patients with a high volume of disease and need of response [12]. It is reasonable to discontinue oxaliplatin or irinotecan after a period of induction therapy (approximately 3–4 months) and continue maintenance single-agent fluoropyrimidine with bevacizumab until the progression of the disease occurs. At that time, reintroduction of the first-line therapy or moving to a second-line therapy can be discussed. After progression on a first-line doublet with bevacizumab, we would change to the alternative second-line doublet while maintaining bevacizumab if it proved to be beneficial in first line [13] (if combined with second-line FOLFIRI, ramucirumab or aflibercept may replace bevacizumab) [14, 15]. If RAS mutation is in K-RAS G12C (approximately 4% of the cases), third-line therapy with a KRAS G12C inhibitor plus an anti-EGFR may be used [16, 17], followed by TFD/TPI and bevacizumab (preferable) or regorafenib on progression [10, 11]. In the case of other RAS mutations, third-line treatment typically involves either TFD/TPI with bevacizumab or regorafenib [10, 11].

For patients with MSS BRAF V600E mutations, although debatable, we tend to offer more aggressive first-line options, such as a triplet with bevacizumab when tolerable [12]. However, a first-line doublet with bevacizumab is also reasonable. Anti-BRAF agents in combination with anti-EGFR are currently offered in the second or later-lines setting, although they are currently being studied as first-line options. It is important to mention that the addition of binimetinib to cetuximab and vemurafenib also yielded benefit when compared to ireinotecan-based chemotherapy and cetuximab in the phase III BEACON trial. However, we prefer to offer cetuximab and encorafenib since this combination performed similarly to encorafenib, cetuximab and binimetinib [9]. For those who progressed both on first-line triplet with bevacizumab and on second-line anti-EGFR plus anti-BRAF, third-line options include either TFD/TPI with bevacizumab (preferable) or regorafenib [10, 11]. For those who used a doublet in the first line setting, the alternative doublet may also be used before the oral drugs.

When the patient has an MSS, RAS and BRAF wild-type tumour, the next important question to answer is the location of the primary lesion. For right-sided tumours, a doublet with bevacizumab is typically the first-line option followed by the alternative doublet with bevacizumab (or other anti-angiogenic) on progression [18, 19]. In these cases, an anti-EGFR with or without irinotecan is used in the third-line setting, followed by TFD/TPI with bevacizumab or regorafenib in later lines for human epidermal growth factor receptor 2 (HER2)-negative tumours and by anti-HER strategies for HER2-positive tumours. Many anti-HER2 agents in combinations have been studied in mCRC, but so far, there is no gold standard [20, 21]. There is even evidence for trastuzumab-deruxtecan or tucatinib with trastuzumab after failure to other anti-HER2 agents and that latter would be our choice of treatment for metastatic HER + CRC, when available [22, 23].

On the other hand, left-sided tumours should be treated with a doublet with an anti-EGFR in the first-line setting [18, 19]. On progression, the alternative doublet with bevacizumab should be used. The third-line option depends on HER2 status and on clinical and/or liquid biopsy-driven results in order to decide whether rechallenge with chemotherapy with anti-EGFR therapy could be useful. If HER2 positive, we suggest anti-HER2 strategies [20–22]. If negative, we would look into progression-free survival on first-line doublet with anti-EGFR. Those patients who benefited from anti-EGFR therapy and, on progression, stayed at least 4 months away from anti-EGFR in the second-line setting, could receive rechallenge with chemotherapy plus an anti-EGFR agent, especially if liquid biopsy rules out RAS mutations (or other anti-EGFR resistance alterations) [24, 25]. However, if those criteria cannot be fulfilled and HER2 is negative, we prefer TFD/TPI with bevacizumab (preferable) or regorafenib as a third-line therapy. More recently, fruquintinib has demonstrated activity in the refractory setting, even after failure to TFD/TPI plus bevacizumab and/or regorafenib, regardless of the molecular profile, and may be an option when available [26].

Neurotrophic tyrosine receptor kinase (NTRK) fusions, although very rare among mCRC, is also a target. NTRK inhibitors, such as larotrectinib, may be used after at least one prior line of therapy for those patients who harbour NTRK fusions, which is more commonly seen in MSI-H tumours without RAS or BRAF mutations [27]. In addition, rearranged during transfection (RET) fusions may also be targeted by selpercatinib [28].

## Conclusion

With this algorithm, GTG believes that most scenarios of unresectable mCRC in fit patients are covered and have the potential to help clinicians in therapeutic decisions. However, access and resource limitations must be considered in clinical practice.

## Figures and Tables

**Figure 1.**
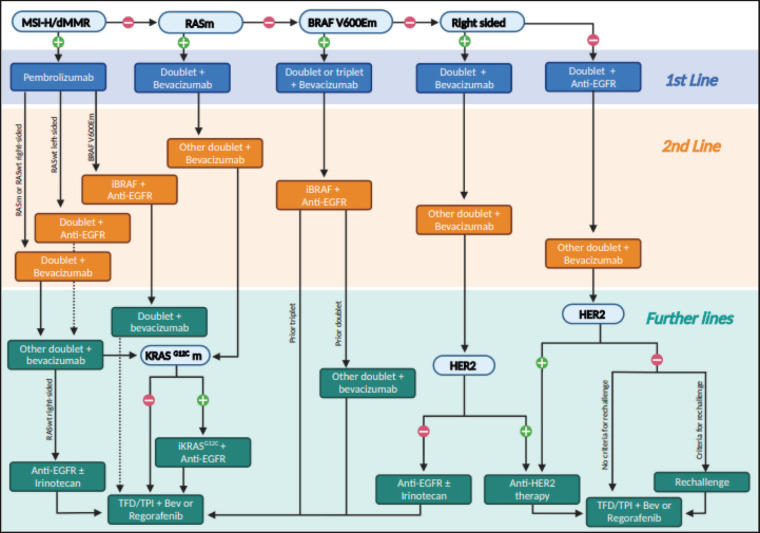
An algorithm for treatment decisions in unresectable mCRC for each line of therapy.
